# Bioinformatic-based Study to Investigate the Structure and Function of Pro-inflammatory Cytokines TNFα and IL-6 Involved in the Pathogenesis of COVID-19

**DOI:** 10.30699/IJP.2024.2015557.3211

**Published:** 2024-02-15

**Authors:** Seyedeh Elham Norollahi, Kosar Babaei, Vida Balooei, Seyed Masoud Hashemi Karouei, Mohammad Taghi Ashoobi, Elahe Asghari Gharakhyli, Ali Akbar Samadani

**Affiliations:** 1 *Cancer Research Center and Department of Immunology, Semnan University of Medical Sciences, Semnan, Iran*; 2 *Noncommunicable Diseases Research Center, Neyshabur University of Medical Sciences, Neyshabur, Iran*; 3 *Department of Veterinary Medicine, Babol Branch, Islamic Azad University, Babol, Iran*; 4 *Department of Surgery, School of Medicine, Razi Hospital, Guilan University of Medical Sciences, Rasht, Iran*; 5 *Department of Biology, Gorgan Branch, Islamic Azad University, Gorgan, Iran*; 6 *Guilan Road Trauma Research Center, Guilan University of Medical Sciences, Rasht, Iran*

**Keywords:** Bioinformatics, COVID-19, Cytokine, IL-6, TNF-a

## Abstract

**Background & Objective:**

Besides the clinical and laboratory research on the COVID-19 virus, the bioinformatics study in the field of genetics of immunity to COVID-19 is of particular importance. In this account, studies show that in patients with COVID-19, the level of tumor necrosis alpha (TNFα) and interleukin-6 (IL-6) is high and in severe cases of COVID-19, the production of IL-6, TNF-α, and other cytokines increases profoundly. On the other hand, investigating the molecular structure and receptors of IL-6 and TNFα and the structural analysis of the receptor proteins may potentially help to develop new therapeutic plans for COVID-19 infection.

**Methods:**

To identify genes with significant and different expressions in patients with COVID-19 in a microarray data set containing transcriptional profiles from GEO as a functional genomic database the GEO query package version 2.64.2 in a programming language R version 4.2.1 was downloaded. In this way, functional enrichment analysis for DEGs, WikiPathways, REGO, gene ontology, and STRING database was also investigated and employed.

**Results:**

The structure and function of pro-inflammatory cytokines TNFα and IL-6 involved in the pathogenesis of COVID-19 were investigated, and in general, after performing various analyses in this study and extracting A series of genes with different expressions from the KEGG database, the final 5 DEGs include CXCL14, CXCL6, CCL8, CXCR1, TNFRSF10, and the relationship and expression effects of them were observed in different pathways.

**Conclusion:**

IL-6 and TNFα were involved in immunological processes that had a direct and indirect relationship with the activation of cytokines, including IL6 and TNF-a, and cytokine storm, and this indicates their role in the formation of problems and complications, including ARDS, in COVID-19 patients. Of course, determining the effectiveness of each of these genes requires more specialized and clinical studies.

## Introduction

COVID-19 is a β-coronavirus caused by infection with SARS-CoV-2. Positive-sense RNA viruses of Corona are involved in the infection of many species, including humans, some mammals, and birds. This virus has the largest genome (26.4-31.7 kilobases) and has a G+C percentage of 32 to 43. Their genome has a poly-A tail. Multiple copies of the nucleocapsid protein inside the virion enclose the genome. In this way, after being infected with this virus, the host may suffer from respiratory, intestinal, liver, and neurological problems (1). In the short time since the emergence of COVID-19, several studies have reported abnormal levels of cytokines and chemokines, including IL-1, IL-2, IL-4, IL-6, IL-7, IL-10, IL-12, IL-13, IL-17, M-CSF, G-CSF, GM-CSF, IP-10, IFN-γ, MCP-1, MIP1-α, hepatocyte growth factor (HGF), TNF-α and growth factor reported vascular endothelial growth factor (VEGF) (2-4). Investigations show that the levels of tumor necrosis alpha (TNFα) and Interleukin (IL-6) are high in patients with COVID-19 And in severe cases of COVID-19, the production of IL-6 and TNF-α and other cytokines is profoundly increased. In addition, patients requiring ICU admission have higher levels of IL-6, IL-2, IL-7, IL-10, GCSF, IP10, CCL2, MIP1A, and TNFα than patients not requiring ICU admission, these results indicate that the cytokine storm is important in the pathogenesis of COVID-19 (5-7). Interleukin-6 (IL-6) is a pro-inflammatory cytokine and bioactive protein and can be produced by different types of immune system cells and also by some non-immune cells such as fibroblasts. The anatomical distribution of IL-6 has been determined in the lungs, urinary bladder, adipose tissue, muscles, and vermiform appendix. IL-6 is recognized by the membrane receptor (IL-6R), which forms a complex with glycoprotein 130 (gp130). This receptor has tyrosine kinase activity and activates signal transducer and activator of transcription 3 (STAT3) through phosphorylation. While the actual function of IL-6 signaling depends on cell type and receptor type, the arrangement of the IL-6 IL-6R axis can be functionally variable. While the interaction of IL-6 with IL-6R and gp130 through the membrane participates in anti-inflammatory signaling, the interaction of IL-6 with IL-6R and gp130 stimulates inflammation (8). IL-6 production is associated with different types of cells such as macrophages, dendritic cells, mast cells, fibroblasts, endothelial cells, and T and B cells. IL-6 plays an important role in regulating many physiological functions such as the cardiovascular system, central nervous system, immune system, etc. High levels of IL-6 and abnormal activation of the IL-6 IL-6R axis are associated with severe disease progression and may be responsible for treatment failure and ultimately fatal complications. A detailed understanding of the biology of IL-6, the IL-6R receptor, and its signaling axis can provide new information to improve the problems of inflammatory diseases and provide an efficient treatment for malignancies and viral infections. A panel of therapeutic antibodies affecting IL-6 signaling is available, but their use has various biological and economic limitations. Nevertheless, in-depth knowledge of the biology of IL-6 signaling along with the precise determination of the relationship between the chemical structure of the inhibitor and the IL-6 IL-6R complex are prerequisites for its rapid addition to therapeutic methods (9-13). Increased levels of TNF-α, as an important pro-inflammatory cytokine, are associated with increased mortality from COVID-19. In patients with rheumatoid arthritis, TNF blockade biologically reduces not only TNF but also other pro-inflammatory cytokines that are important in the hyper-inflammation of COVID-19. Data from patients previously treated with anti-TNF therapy showed poorer COVID-19 outcome rates and reduced mortality compared with other immunosuppressive regimens (14). The biology of TNF-α has properties that make it a promising target for treatment in patients with COVID-19. Its role in inflammatory disease has been extensively studied for more than 20 years, and the relative efficacy and safety of anti-TNF in many diseases have led to major changes in medical science. TNF-α plays an important role in the initiation of the inflammatory cascade and increases early in the disease process. Accordingly, early intervention may be more beneficial. This coordinates cell recruitment through the regulation of chemokines and adhesion molecules. Anti-TNF therapy also results in reduced production of other pro-inflammatory cytokines, including IL-1 and IL-6. Therefore, inhibition of TNF-α in patients with COVID-19 may reduce many of these pathogenic cytokines (15, 16). Considering that the approved treatments are still very limited, informatics-based studies provide new, efficient, and testable hypotheses in viral investigations to recognize the virus and the systematic use of drugs in the treatment process (17). Effective measures against the COVID-19 virus require the development of data and tools to understand and monitor the outbreak and respond appropriately. Also, the significant increase in the number of coronaviruses and their genomes made it possible for specialists to quickly perform diagnostic tests for the virus through genetic sequence checks and copy synthesis (18, 19). Therefore, investigations of the involved and influential genes give us a good opportunity to perform bioinformatics and genomic analyses in this family of viruses. On the other hand, investigating the molecular structure and receptors of IL-6 and TNFα and the structural analysis of the ligand/receptor proteins may potentially contribute to the development of new therapeutic plans because these molecules are currently considered the main targets in the cytokine storm of COVID-19 and the pathogenesis of acute respiratory distress syndrome are considered. However, in this study, we determined the molecular structure and receptors of IL-6 and TNFα alongside the function of pro-inflammatory cytokines TNFα and IL-6 involved in the pathogenesis of COVID-19.

## Material and Methods

**Study Plan**

The study initially was confirmed by the ethical committee of Azad University of Babol Branch, Iran (Ethical code: IR.IAU.BABAOL.REC.1400.144) and then the required methods and databases were employed.

**Review and Analysis of GEO Data**

The Gene Expression Omnibus (GEO) database is an international public database that archives and freely distributes high-throughput gene expression and other functional genomic datasets. GEO supports community-derived reporting standards that specify the provision of several important study elements, including raw data, processed data, and descriptive metadata. The database not only provides access to data for tens of thousands of studies but also provides a variety of web-based tools and strategies that enable users to find data relevant to their specific interests, as well as review and analyze the data. The GEO home page is at (http://www.ncbi.nlm.nih.gov/geo/) (20).

To identify genes with significant and different expressions in patients with COVID-19 in a microarray dataset containing transcriptional profiles from GEO as a functional genomic database using the GEOquery package version 2.64.2 in a programming language. R version 4.2.1 was downloaded. In this study, the datasets GSE164805 with GPL26963 were used, which contain three types of patient samples: severe, mild, and healthy.

Gene expression technologies are often used in molecular biology research to obtain a snapshot of transcriptional activity in different tissues or populations of cells. These profiles are then compared to identify gene expression changes associated with the treatment condition or phenotype of interest. Over the past decade, limma has been a popular choice for gene discovery through differential expression analysis of microarray data and high-throughput PCR. This package includes very powerful facilities for reading, normalizing, and exploring such data.

The limma package based on the Bioconductor package was used to analyze the microarray data to determine differentially expressed genes (DEGs) (21). These criteria log2fold-change > 3 and Adj.P.Val < 0.05 were considered statistically significant.

**Functional Enrichment Analysis for DEGs**

Functional enrichment analysis is a method to identify classes of genes or proteins that are over-represented in a large set of genes or proteins and may be associated with disease phenotypes. This method uses statistical approaches to identify enriched or depleted groups of genes. Transcription technologies and proteomics results often identify thousands of genes that are used for analysis. 

The signaling pathways related to the gene list under our study are examined with the existing lists in this database in different pathways. Correspondingly, the KEGG pathway is the most important and reliable in examining gene data. In this study, WikiPathway, KEGG (Kyoto Encyclopedia of Genes and Genomes), and GO (Gene ontology) were used to discover the main function and information of enriched pathways related to DEGs.

**WikiPathways**

WikiPathways (https://www.wikipathways.org) is an open and collaborative platform dedicated to the study of biological pathways, established to facilitate the contribution and maintenance of pathway information by the biology community. Thus, WikiPathways provides a new model for pathway databases that will enhance and complement current efforts such as KEGG, Reactome, and Pathway Commons (22).

**KEGG**

KEGG (Kyoto Encyclopedia of Genes and Genomes) is a database resource for understanding high-level functions and applications of biological systems, such as cells, organisms, and ecosystems, from molecular-level information, especially large-scale molecular datasets generated by genome sequencing and other Experimental technologies are produced with high efficiency (23).

**Gene Ontology**

Gene Ontology (GO; http://www.geneontology.org) is a community-based bioinformatics resource that provides information on gene product function using ontologies to represent biological knowledge. This knowledge is both human-readable and machine-readable and is the basis for computational analysis of large-scale molecular biology and genetics experiments in biomedical research (24).

To check the pathways related to DEGs in the wikipathway database, the clueGO plugin was used in the Cytoscape software, and the clusterProfileR package in the R programming language was used for KEGG and GO.

**STRING Database**

Cellular life depends on a complex network of functional connections between biomolecules. Among these connections, protein-protein interactions are of particular importance due to their versatility, specificity, and compatibility. STRING (https://string-db.org/), an online search tool for predicting protein-protein interactions (PPI), is used to construct a PPI network. These interactions include direct (physical) and indirect (functional) communication. They originate from computational prediction, knowledge transfer between entities, and interactions collected from other (primary) databases. The STRING database currently covers 24584628 proteins from 5090 organisms (25). In this study, we used the string plugin in Cytoscape software.

## Results

**GEO Data Analysis Results**

**Fig. 1:**
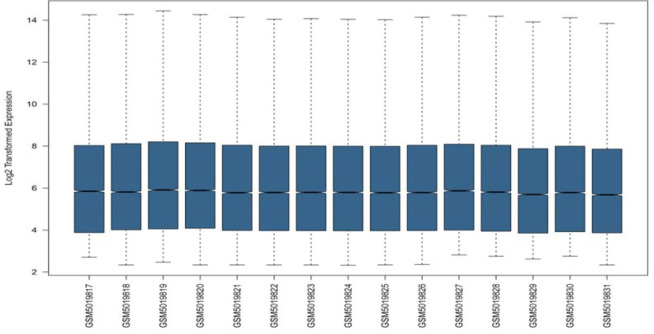
Boxplot of normalization of microarray data from the whole genome transcript of peripheral blood mononuclear cells of 10 samples of COVID-19 patients and 5 healthy samples.

To investigate the pattern of gene expression changes and microarray analysis of the whole genome transcriptome on peripheral blood mononuclear cells (PBMCs) taken from severe and mild COVID-19 patients as well as healthy individuals, Array preprocessed expression data from the GEO site via The GEOquerry package was downloaded in R software and adjusted with commands and codes and analyzed based on the determined criteria. First, the raw data were examined in terms of normalization, which was normalized to our GSE164805 dataset (Figure 1).

**Fig. 2:**
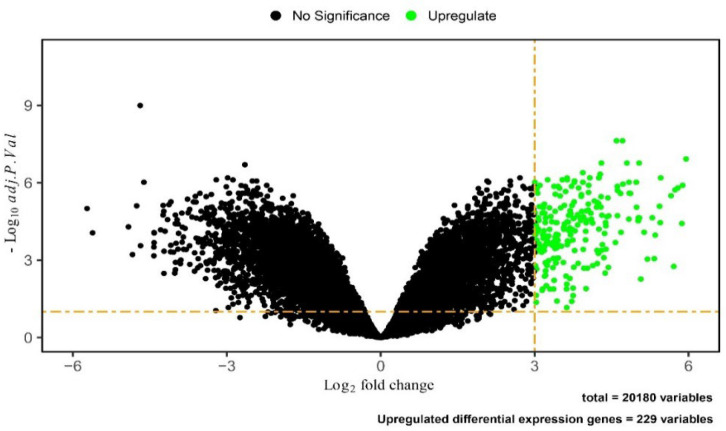
Volcano plot of 229 DEGs from the data of COVID-19 using R software.

We used the limma package to analyze all microarray data and obtained a string of statistically significant DEGs consisting of 44,825 variables. In this way, 728 genes with different expressions from other genes were identified as main DEGs using adj.P-value <0.05 and log2fold-change > 3 criteria. This set of DEGs was identified in the COVID-19 data compared to normal samples, 229 of which have increased expression (Figure 2).

**WikiPathway Enrichment Analysis for DEGs**

**Fig. 3:**
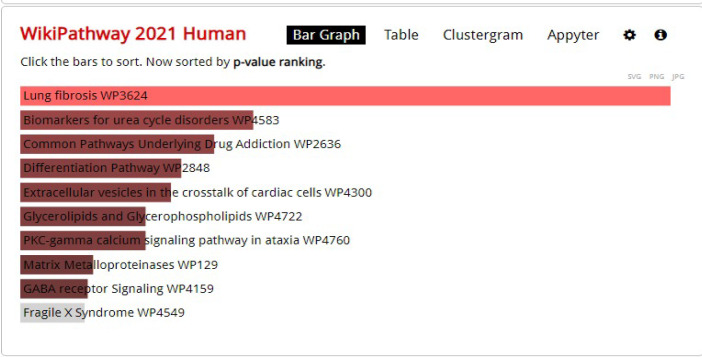
Review of the general biological pathways of 229 DEGs focusing on metabolites and their relationship with diseases from WikiPathway in the Enrichr database.

**Fig. 4:**
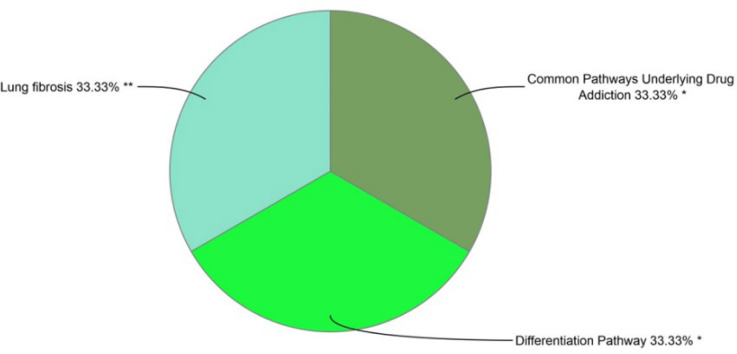
Pie chart using clueGO plugin in Cytoscape software.

WikiPathway in the Enrichr database was used to investigate the general biological pathways of these 229 DEGs, focusing on metabolites and their relationship with diseases (Figure 3). Correspondingly, also using the clueGO plugin in Cytoscape software was used to draw a pie chart (Figure 4).

The results of WikiPathway related to DEGs showed that these genes have the greatest impact on the pathways related to lung fibrosis. It can be an indication of the great impact of these proteins on COVID-19.

**KEGG Pathway Enrichment Analysis for DEGs**

**Fig. 5:**
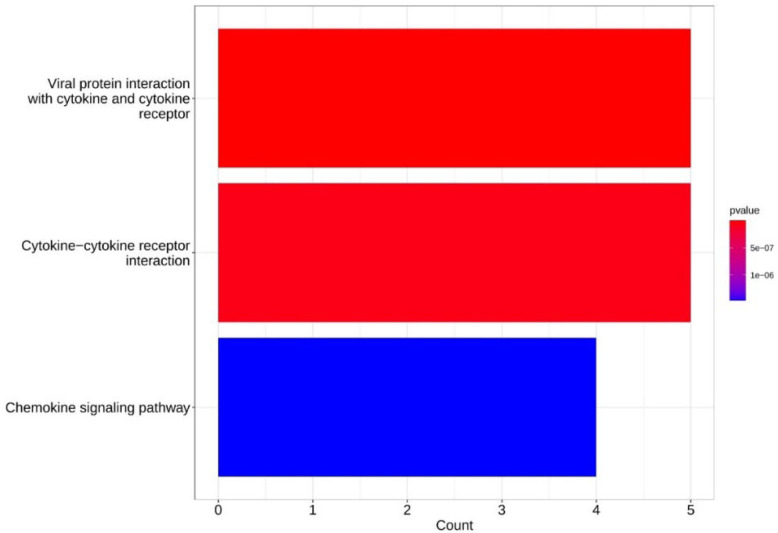
KEGG analysis on 229 DEGs using Clusterprofiler package.

KEGG analysis was performed on DEGs using the Clusterprofiler package, to investigate the biological pathways of 229 DEGs (Figure 5).

The first 9 pathways of the analysis of these 229 DEGs in the KEGG database are shown in Table 1.

**Table 1:** The first 9 paths of the analysis of these 229 DEGs are in the KEGG database.

Count	Gene ID	Q value	p. adjust	P-value	Bg Ratio	Gene Ratio	Description	ID
5	9547/6372/6355/3577/8794	0.626019	0.639724	0.004004	100/8146	May-86	Viral protein interaction with cytokine and cytokine receptor	hsa04061
4	9474/3479/8660/64764	0.626019	0.639724	0.014326	89/8146	Apr-86	Longevity regulating pathway	hsa04211
2	23205/55301	0.626019	0.639724	0.015105	18/8146	Feb-86	Fatty acid biosynthesis	hsa00061
6	1991/10912/7102/3479/4318/7850	0.626019	0.639724	0.016056	193/8146	Jun-86	Transcriptional misregulation in cancer	hsa05202
4	3479/4318/7850/64764	0.626019	0.639724	0.019085	97/8146	Apr-86	Prostate cancer	hsa05215
5	56901/4696/140679/2561/2911	0.626019	0.639724	0.019869	148/8146	May-86	Retrograde endocannabinoid signaling	hsa04723
4	5052/5273/7850/383	0.626019	0.639724	0.022503	102/8146	Apr-86	Amoebiasis	hsa05146
3	9474/3479/8660	0.669068	0.683715	0.027486	62/8146	Mar-86	Longevity regulating pathway - multiple species	hsa04213
7	9547/6372/3596/6355/3577/8794/7850	0.687607	0.70266	0.035814	295/8146	Jul-86	Cytokine-cytokine receptor interaction	hsa04060

The relevant enrichKEGG codes in the R programming language and statistical analysis criteria were used. In this way, p-value ≤ 0.05 in the KEGG database showed that 5 DEGs play a very important role in the interaction of viral proteins with cytokines. According to the geneIDs in the table and the information extracted from the KEGG database, these 5 DEGs include CXCL14, CXCL6, CCL8, CXCR1, TNFRSF10.

**Construction of Protein-Protein Interaction Network (PPI)**

**Fig. 6:**
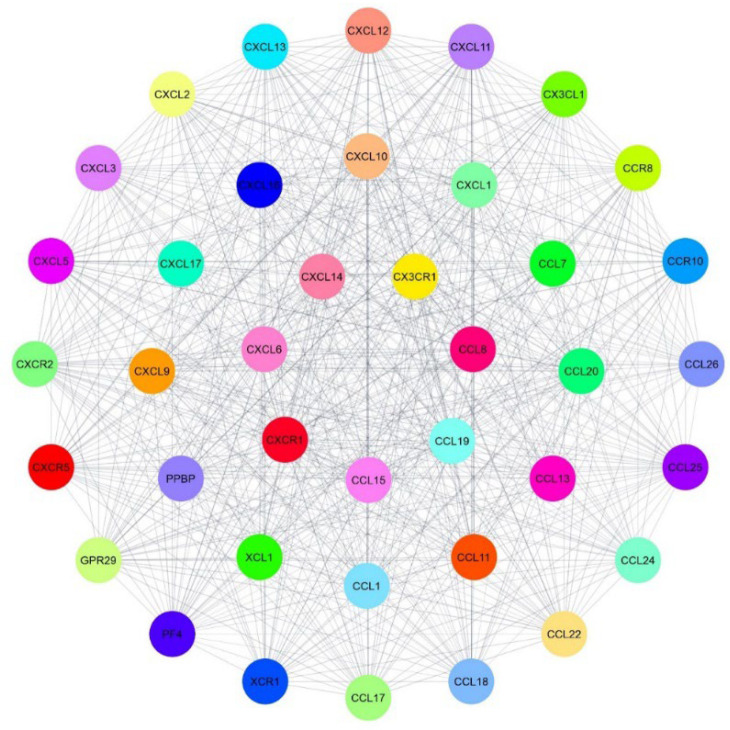
Interaction of 35 other proteins with 5 DEGs inside the STRING database.

First, we entered the STRING database, and then we entered the names of 5 DEGs obtained from KEGG, which were related to the interaction of viral proteins with cytokines, into the multiple proteins search. The results showed that 35 other proteins have the most interaction with 5 DEGs obtained in the STRING database (Figure 6).

**GO and KEGG Pathway Enrichment Analysis for Proteins Resulting from the PPI Network**

**Fig. 7:**
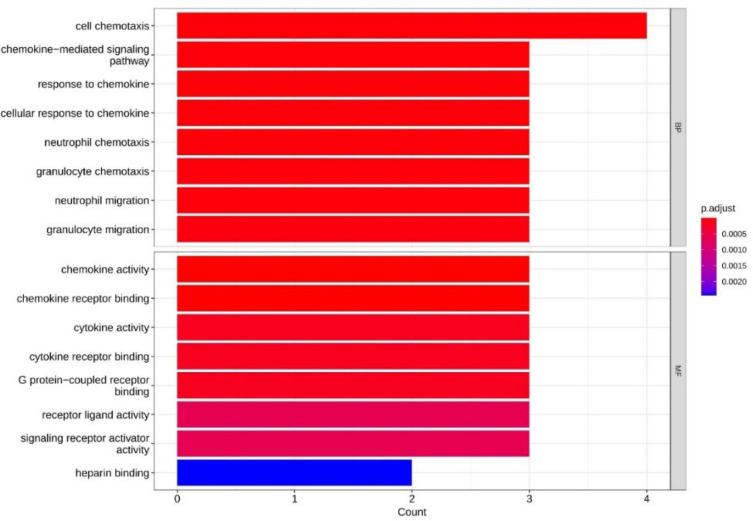
Ontology examination of genes with different and effective expression.

The genes obtained from the STRING site, which had significant differential expression after meta-analysis, were entered into the GO database in the form of a gene list so that by comparing their ontology with the list of genes classified in this database, a view can be obtained (Figure 7). In general, ontology in biological, cellular, and molecular dimensions examines genes with different expressions obtained from its data.

By carrying out the ontology process, the results of the investigation showed the role of the obtained genes in the following pathways, including the signaling pathway mediated by chemokines and cytokine storm.

**Fig. 8:**
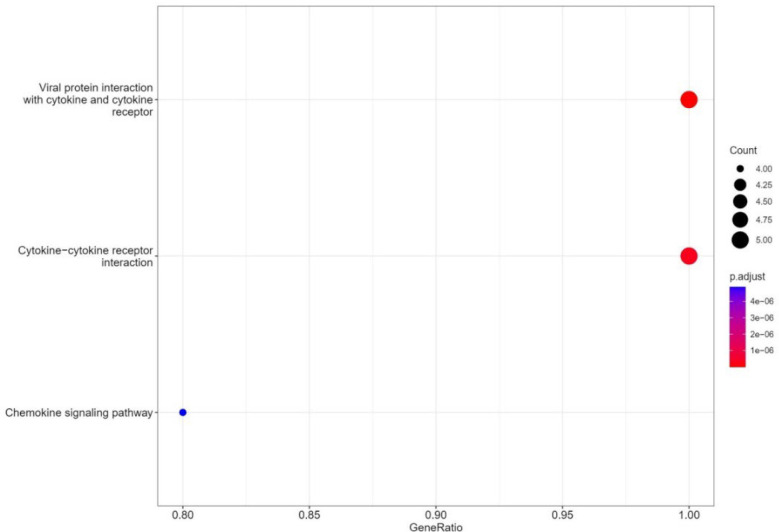
KEGG path analysis of the studied genes with different and effective expressions.

The KEGG pathway examination was also almost in line with confirming the same ontology pathways (Figure 8).

**Fig. 9:**
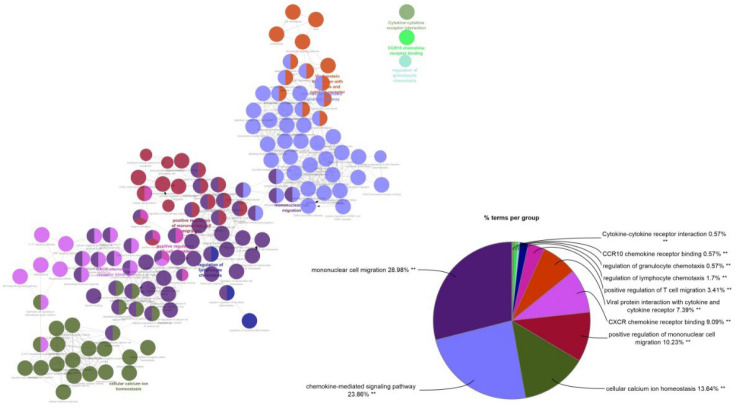
Checking the interaction between Go and KEGG using the clueGO plugin in Cytoscape software.

In addition to the clusterprofiler package, the R programming language for Gene ontology and KEGG was checked, the clueGO plugin in the Cytoscape software was used to check the four categories of Go, including biological process, cellular component, molecular function, and immune system process, along with KEGG (Figure 9).

## Discussion

Further studies of a cell surface protein, known as angiotensin-converting enzyme II (ACE2), have been identified to be involved in receptor-mediated endocytosis for the entry of SARS-CoV-2 into cells (26). And many studies have reported the clinical features of COVID-19. In a COVID-19 outbreak, about 1-2 weeks after onset, some infected patients suddenly worsen and develop acute respiratory distress syndrome (ARDS), followed by shock, late-stage tissue perfusion disorders, and finally due to Multiple organ failure and its exacerbation mainly caused by very high pro-inflammatory cytokines (cytokine storm), very low levels of lymphocytes, especially natural killer (NK) cells in the peripheral blood, and C-reactive protein (CRP)(27). The severe clinical phase of COVID-19 is associated with high levels of cytokine signaling, therefore, identifying how to prevent the cytokine storm and when to initiate anti-inflammatory therapy are crucial to reducing the mortality rate of COVID-19(28). In a 2020 study aimed at understanding the cytokine storm, Hu and colleagues investigated the pathological features, pathophysiological mechanisms, and treatment of the cytokine storm caused by COVID-19 and concluded that the cytokine storm leads to harmful clinical manifestations or even death. Acute mortality in critically ill patients with COVID-19 Impaired acquired immune responses and uncontrolled innate inflammatory responses may be related to the cytokine storm mechanism in COVID-19. They also pointed out the importance and level of IL6 and TNF-a during the disease and their role in the cytokine storm (29). In 2020, Coomes et al conducted a systematic review and meta-analysis to evaluate interleukin-6 (IL-6) levels in complicated cases of COVID-19. In this study, they showed that serum levels of IL-6 are significantly increased in the setting of complicated disease of COVID-19 and that elevated levels of IL-6, in turn, are significantly associated with adverse clinical outcomes. This suggests that the progression of primary SARS-CoV-2 infection to complex disease may be the result of an excessive host immune response and autoimmune damage resulting from a cytokine storm(30). In a 2020 study targeting inflammation and cytokine storm in COVID-19 by Huang and Wu indicated that patients with severe COVID-19 had higher levels of cytokines than patients with moderate symptoms. Inflammatory factors, such as IL-1β, IL-6, IL-10, and serum d-dimer, show the involvement of a cytokine storm. Cytokine storm represents an exaggerated immune response characterized by the overproduction of proinflammatory cytokines and chemokines such as IFN-γ, TNF-α, IL-6, IL-1β, IL-18, CXCL8, and CXCL10. While the adequate release of cytokines is critical for the body's defense against viral infection, uncontrolled and aberrant activation of the immune system can lead to organ damage. Current clinical evidence, as well as the association of cytokine storm syndrome with disease severity, supports its adverse outcomes in hospitalized COVID-19 patients(5, 31). Since the current management of COVID-19 is supportive and respiratory failure due to acute respiratory distress syndrome (ARDS) is the main cause of death(32).The association of unfavorable prognosis with excessive presence of inflammation and cytokine storm highlights the necessity of early detection of cytokine storm and implementation of anti-inflammatory therapy. Timely control of the hyperinflammatory response is critical to prevent the development and progression of irreversible ARDS and multiorgan dysfunction associated with COVID-19. Today, with technological advances in bioinformatic processes, researchers have been granted access to study thousands of genes at the same time in different pathogens. Based on this and by using some of these bioinformatics tools, we were able to identify a large number of protein-coding genes in the human genome that are constantly involved in the PBMC (peripheral blood mononuclear cell) of COVID-19 patients and have important effects in the pathogenesis of the signaling pathways of the factors involved in this disease. Here we have performed a comprehensive analysis of DEGs and compared three groups of human subjects using advanced methods and statistical techniques. The dataset we used for the study (GSE164805) includes fifteen human subjects (five controls, five mild COVID-19 patients, and five severe COVID-19 patients). The data set is limited because it was only collected from PBMC samples from three groups of human subjects (controls, mild COVID-19 patients, and severe COVID-19 patients). A similar data set is not available in the database that uses PBMC for its analysis of COVID-19 patients and compared to controls. It was noted that this dataset was only the first dataset in the GEO database to collect gene expression data from three cohorts of human subjects. Gene expression data were analyzed using a microarray platform. This dataset was originally submitted to the database early (January 2021), when no gene expression data were available from all three groups of COVID-19 patients. From this point of view, the dataset is very important. We obtained the upregulated DEGs from the corresponding dataset using the R programming language. We believe that similar studies with more patient datasets from other parts of the world will significantly increase our understanding of this complex virus-host interaction during the progression of the COVID-19 disease and help to map the genes involved in protective immunity. We performed functional pathway enrichment analysis to understand the up-regulated genes of COVID-19 and target genes. We reported several genes that are upregulated in COVID-19 and target genes related to immune cell activation, such as the interaction of viral proteins with cytokine receptors and pathways related to chemokines and cytokines. Our co-expression network pattern of protein-coding DEGs revealed the prospective function of differentially expressed genes in the context of COVID-19. In 2020 dhall et al., studied the goal of computer-aided prediction and design of IL-6-inducing peptides in COVID-19. IL-6 plays a critical role in the progression of COVID-19 and is responsible for the high mortality rate. In order to facilitate the community to fight against COVID-19, in this study, a method was developed to predict IL-6 inducible peptides/epitopes. These models were tested on 365 IL-6 inducible and 2991 non-inducible peptides extracted from the immune epitope database. Initially, 9149 features of each peptide were calculated using Pfeature, which was reduced to 186 features using the SVC-L1 method. These features were ranked based on their classification ability and the top 10 features were used to develop predictive models. A wide range of learning techniques has been used to develop models. Also, using the best models provided for designing a vaccine against COVID-19, IL-6-inducing peptides have been identified in different SARS-COV2 proteins(33). In 2020, Zhou et al. investigated the relationship of translated proteins of 2019-nCoV with other Orthocoronavirinae species through bioinformatics-based methods. Phylogenetic analysis of all 15 HCoV genomes that 2019-nCoV/SARS-CoV-2 shares the most nucleotide sequence similarity with SARS-CoV. Overall, the envelope and nucleocapsid proteins of 2019-nCoV/SARS-CoV-2 are conserved regions that have 96 and 89.6% sequence similarity compared to SARS-CoV, respectively. By using network analyses of drug targets and interactions of the coronavirus and the host in humans, 16 possible anti-coronavirus drugs such as melatonin, mercaptopurine, and sirolimus, based on their gene-drug effect studies and transcript data caused by the coronavirus in Confirmed human cell lines were prioritized. Also, a combination of three potential drugs including sirolimus + dactinomycin, mercaptopurine + melatonin, and toremifene + emodin was identified. this study presented network-based methods for the rapid identification of reversible drugs and potential drug combinations targeting nCoV/SARS-CoV-2. In this way, in 2020, Cao et al. conducted a comparative analysis of the ACE2 receptor genetics of the novel coronavirus (nCoV/SARS-CoV-2) in different populations. They systematically analyzed ACE2 coding region variants and eQTL variants that may affect ACE2 expression using the GTEx database to compare ACE2 genomic features among different populations. The findings showed that the genetic evidence of the presence of resistant ACE2 mutations to bind the S protein of the coronavirus has not been detected in different populations. The obtained data can contribute to further research on ACE2, including its role in acute lung injury and lung function. The East Asian population has a higher allele frequency in eQTL variants with higher expression of ACE2 in the tissue; This shows that the sensitivity and response of the coronavirus are different in different populations under the same conditions. Remarkably, Grifoni et al compiled a known epitope sites from other coronaviruses to map the responsible regions in the SARS-COV2 sequence and predict its possible epitopes. For this purpose, they used IEDB and ViPR databases. The IEDB contains SARS-CoV, which is very similar to the sequence of SARS-COV2 and is the best-characterized coronavirus in epitope responses. Also, valid bioinformatics tools were used to predict B and T cell epitopes that are most likely known in humans and to evaluate the degree of protection of these epitopes in different types of coronaviruses. The independent identification of similar regions shows that these results were used as a promising target for the diagnosis of SARS-COV2 immunity. These predictions can also facilitate the design of an effective vaccine against this virus. In a study by Tang, molecular patterns between SARS-COV2 and other coronaviruses were investigated. Although genomic analyses suggested that SARS-COV2 is closely related to RaTG13, their differences in neutral regions are much greater than previously found. The results of this study provide new insight into the natural host for SARS-COV2. By examining the population genomics of 103 SARS-COV2 genomes, they found that SARS-CoV-2 viruses evolved into two main types, L and S, and these two types are defined by only two SNPs, which are completely related to SARS-CoV-2 strains. Although the prevalence of type L is 70% higher than type S, and their findings indicated that type S is likely to be an older version of SARS-CoV-2. Also, the results of this study showed that type L is more aggressive than type S(34). In another study by Woo PC and colleagues, the current findings in the bioinformatics and genomic analysis of the coronavirus were reviewed. Since coronaviruses are propagated from different samples, they may contain the same genes or genomic sequence, which leads to difficulty in analysis. Due to these problems, in this study, a comprehensive CoVDB database of genes and The genome of the coronavirus is presented, which enables fast and efficient sequence analysis. In CoVBD, the interpretation of non-structural proteins in polyproteins coded by ORF1ab from each unit sequence and proteins and downstream of ORFs in ORF1ab was done through the standard system. All sequences are labeled with specific nucleotides and indicate strains with specific sequences (35). In addition to the complete sequence of the coronavirus genome, this database also includes their incomplete genomes and genes. This is very useful because many genes of this virus such as Pol, S, and N have been sequenced more than others because they are more or less protected, so it is of special importance for primer design in RT-PCR assays and evolutionary studies. A study investigated the multi-platform omics analysis of serial plasma and urine samples collected from patients during the COVID-19 period. Integrated analysis of these omics data revealed several potential therapeutic targets, such as ANXA1 and CLEC3B. Molecular changes in plasma indicated dysregulation of macrophages and suppression of T cell function in severe patients compared to non-severe patients. In addition, they selected some important molecular markers as potential biomarkers for predicting disease severity (36). The predictive power was confirmed using the corresponding urine samples and plasma samples from a cohort of new COVID-19 patients, with an AUC of 0.904 and 0.988, respectively. In conclusion, omics data suggest not only potential therapeutic targets but also biomarkers for understanding the pathogenesis of severe COVID-19. A study on metabolomics and cytokine/chemokine profile on serum samples from healthy individuals and mild and severe COVID-19 patients was done. Correlation analyses revealed a close relationship between metabolites and pro-inflammatory cytokines/chemokines, such as IL-6, M-CSF, IL-1α, and IL-1β, suggesting a potential regulatory interaction between arginine, tryptophan, purine metabolism, and hyperinflammation (37). Importantly, targeting metabolism significantly modulates the release of proinflammatory cytokines by peripheral blood mononuclear cells isolated from a cohort of primates infected with SARS-CoV-2, suggesting that exploiting metabolic changes may be possible. A potential strategy for the treatment of fatal CRS in COVID-19. Importantly in a related study, the immunopathogenesis and immunogenetic variants involved in COVID-19 have been investigated. In this research, they reviewed the understanding of the immunogenetic etiology and pathophysiology of COVID-19 and the related cytokine storm and constructed and analyzed protein-protein interaction (PPI) networks (using enrichment and annotation analysis) based on types NLRP3 and IL18 and all genes involved in severe COVID-19(38). They showed that PPI network and enrichment analyzes identified useful drug targets to prevent the onset of severe COVID-19 including key antiviral pathways such as Toll-like receptor cascades, NOD-like receptor signaling, interferon RIG induction ( IFN) α/β, and interleukin (IL)-1, IL-6, IL-12, IL-18, and tumor necrosis factor and also innate immune evasion of SARS-CoV-2 and involvement of MYD88 and MAVS in the pathophysiology of COVID-19. Also, genetic variants of the PPI network may be used to predict more severe outcomes of Covid-19, thus opening the door to targeted preventive therapies. Conclusively, these essential DEGs may serve as potential biomarkers for COVID-19. We described and categorized the function of genes related to the regulation of immune and protective immunity elements by analyzing DEGs in COVID-19 and provided a list of genes regulating the immune system and regulating immune-related transcripts in COVID-19. The present study investigated the role of pro-inflammatory cytokines IL6 and TNF-a in the pathogenesis of Covid-19.

## Conclusion

In general, after performing various analyses in this study and extracting a series of genes with different expressions from the KEGG database, the final 5 DEGs include CXCL14, CXCL6, CCL8, CXCR1, TNFRSF10, and examining their relationships and expression effects in different pathways. It was observed that all of them were involved in different ways and in different ways in immunological processes that have a direct and indirect relationship with the activation of cytokines, including IL6 and TNF-a and cytokine storm, and this indicates their role in the formation of problems. Complications include ARDS in COVID-19 patients. Of course, determining the effectiveness of each of these genes requires more specialized and clinical studies.
